# Current approaches for the management of Parkinson’s disease in Chinese hospitals: a cross-sectional survey

**DOI:** 10.1186/s12883-018-1122-4

**Published:** 2018-08-22

**Authors:** Gang Wang, Hai-Lun Cui, Jun Liu, Qin Xiao, Ying Wang, Jian-Fang Ma, Hai-Yan Zhou, Jing Pan, Yu-Yan Tan, Sheng-Di Chen, on behalf of the Chinese Movement Disorders Society

**Affiliations:** Department of Neurology& Institute of Neurology, Ruijin Hospital affiliated to Shanghai Jiaotong University School of Medicine, No.197, Rui Jin Er Road, Shanghai, 200025 China

**Keywords:** Parkinson’s disease, Surveys and questionnaires, Guideline, Disease management

## Abstract

**Background:**

Chinese guidelines for management of Parkinson’s disease (PD) have been issued and updated regularly since 2006. We undertook a cross-sectional survey to evaluate the impact of the latest edition (2014) on current approaches to the management of PD based on previous pilot works.

**Methods:**

Seven hundred and seventeen participants, divided into 3 groups (GPs, Neurologists, and Specialists), recruited from 138 randomly chosen hospitals from 30 cities across China, participated by completing the questionnaire describing their current approaches before and after the guidelines were issued.

**Results:**

Considerable discrepancies in management were apparent across the three categories, with different selection of first-choice medication for PD patients. There were also variations in management of concurrent psychiatric symptoms and dementia. Notably, over 50% of participants reported improvements in PD recognition and management by following the guidelines.

**Conclusions:**

The increasing use of Chinese clinical practice guidelines for PD management is having a positive impact on the optimization of care, which in turn offers important economic benefits.

## Background

Being one of the most common neurodegenerative disease worldwide, Parkinson’s disease (PD), with its high prevalence and incidence among aged people, carries a huge economic burden in China and presents with some unique features in Chinese population [1]. To address the aging crisis, the Chinese Parkinson’s Disease & Movement Disorders Society (CMDS), Neurology Branch of Chinese Medical Association has consecutively issued three versions of management guidelines for PD since 2006 [2–4]. Drawing upon four eminent guidelines globally ahead, which were formulated respectively by the Movement Disorder Society (MDS) [5], the American Academy of Neurology (AAN) [6–9], the European Federation of Neurological Societies (EFNS) [10], and the UK’s National Institute for Health and Care Excellence (NICE) [11]—the CMDS guidelines aim to guide the general direction of PD treatment in order to improve the current management standards in China. A key unique individualized treatment recommendation was the principles of dosage titration and a satisfactory clinical effect with a low dose, i.e., “low and slow”, should be followed to avoid acute adverse reactions and to reduce the incidence of long-term motor complications, as important therapeutic strategies in optimizing and standardizing treatment. Specifically, we recommend the principles of medication should be aiming at an optimal status for working and living quality, rather than full-dosage administration [12].

Aiming to acquire the real situation of PD treatment in China, in 2011 we conducted a pilot survey and the results showed significant differences in treatment options and an obvious variation before and after referring of PD guidelines among GPs, general neurologists and movement disorders specialists [13]. Given the relatively small sample size of enrolled doctors involved previously, we launched a national survey at a larger scale and specify our research on the latest edition of the PD guideline, hoping to update our awareness of current PD management in China and measure the impact of the latest version (2014). Thus, this survey would evaluate the status quo and shed new light on the future control and management of PD.

## Methods

### Recruitment

This cross-sectional survey on doctors was performed between December 2015 and February 2016. One hundred and thirty-eight hospitals of different levels were chosen randomly from 30 cities across China. We enrolled 717 doctors in the survey and classified them into three categories: (1) GPs—most belong to general physicians; (2) General neurologists—who specialize in general neurology other than movement disorders; (3) Movement disorders specialists—neurologists with a specific focus on movement disorders, who regularly attend the movement disorders clinics. Among the chosen hospitals, 96% were ranked as tertiary hospitals according to the current grading measures issued by the PRC National Health and Family Planning Commission, which classes hospitals as tertiary if they are characterized by comprehensive multiple functions with specialization in all-purpose high-quality medical services and academic researches across different areas.

### Research content

We asked enrolled doctors to complete a 96-item questionnaire covering all aspects of PD, including relevant methods on PD management, and the various therapeutic options they offered to patients. The questionnaire was designed by two of the authors (Dr. Gang Wang and Dr. Sheng-Di Chen) with approval from the Research Ethics Committee, Ruijin Hospital affiliated to Shanghai Jiao Tong University School of Medicine, Shanghai, China. The study included three stages, as following:

- Stage1: Background Acquirement: We gathered demographic information from the participants enrolled in the study in order to examine the distribution of the samples. We also assessed the level of understanding on a general basis in order to obtain an overall standard of recognition with regard to PD among the participants.
- Stage 2: Differences in PD therapy according to varied clinicians, levels of hospital and city: We questioned participants with detailed and targeted questions on the different medications they tended to offer PD patients who presented with various symptoms. In order to evaluate the whole situation on a more comparable and meticulous basis, we classified the participants into three categories: by clinicians (GPs/Neurologist/Specialist), level of hospital, and hierarchy of city they worked in (as defined by the general economic status of the city).
- Stage 3: Guideline’s impact on PD recognition and treatment: Finally, we attempted to investigate the disparity in the understanding and therapeutic methods of PD via the intervention of the latest version CMDS Guidelines (2014) and observed the changes in doctors on how they viewed and dealt with PD after adopting the guideline. The comparison of the two phrases (before and after the release of the guidelines) would be beneficial for the future training of the physicians in the management of PD.

### Statistical analysis

All statistics were evaluated and addressed by members of an independent third party, with no underlying competing interests involved. Categorical variables were displayed by frequencies and percentages. The raw data analysis was based on contingency tables. All statistical analyses were performed using SAS version 9.4 software. Chi-square test was used for statistical analysis, and a *P*-value < 0.05 was considered significant.

## Results

### Demographics

A total of 717 (Male: female = 43%: 57%) participants were enrolled and mostly aged between 35 and 60 years (66%). Six hundred eighty-four participants (96.2%) were doctors of tertiary hospitals. As for the clinician types, 54% of them were general neurologists, with 28% of specialists in PD, others (18%) were GPs. Among all, 70.4% had no academic memberships, while 17.9% were members of CMDS in China. We also investigated the general level of CME of PD among physicians from various departments. Notably, there were significant differences (*p* < 0.05) in their familiarity with PD guidelines, exchange of academic experience, and level of research for PD.

### Differences in PD therapy according to various clinicians, hospitals and cities

With each of the three categories, we sought participants’ opinions on the same set of therapeutic measures. Across various clinicians, there was a lack of consensus on several treatment issues, such as the first choice of medication for patients without cognitive decline, management on wearing off phenomenon, peak-dose dyskinesia and morning dystonia (see Table 1). They also thought differently on the management of PD with dementia and/or psychiatric symptoms. When faced with psychiatric symptoms in advanced stage patients, most of the PD specialties (62.0%) would reduce or stop the anti-PD medication, while only 33.08% of GPs would copy the same strategy (χ^2^(*N* = 711) = 26.4027, *p* < 0.0001). However, regardless of their clinician types, most of the participants agreed on the usage of MAO-B inhibitors, which they prescribed for 25–50% of their PD patients to alleviate their symptoms. Nevertheless, there was disagreements about which type of PD might benefit more from MAO-B inhibitors, and an extremely significant disparity (*p* < 0.0001) on familiarity with the use of disease-modifying/neuroprotective therapy (χ^2^(*N* = 711) = 42.9005, *p* < 0.0001), Rivastigmine for PDD patients (χ^2^(*N* = 711) = 20.2759, *p* < 0.0001), and antipsychotics for psychiatric symptoms managements (χ^2^(*N* = 711) = 21.8987, *p* < 0.0001).

**Table 1:** Preferred medication for PD patients under specific circumstances among doctors of different specialties

Items	Total (*n* = 711)^a^	General physicians (*n* = 130)	General neurologists (*n* = 381)	Movement disorders specialists (*n* = 200)	χ^2^	*P* value
Age < 65 years without cognitive impairment						
Levodopa	185 (26.0%)	25 (19.2%)	106 (27.8%)	54 (27.0%)	3.8551	0.1455
Dopamine agonists	333 (46.8%)	38 (29.2%)	177 (46.5%)	118 (59.0%)	28.0886	< 0.0001
MAO-B inhibitors	164 (23.1%)	20 (15.4%)	86 (22.6%)	58 (29.0%)	8.3434	0.0154
Benzhexol	49 (6.9%)	4 (3.1%)	25 (6.6%)	20 (10.0%)	6.0243	0.0492
Amantadine	101 (14.2%)	13 (10.0%)	55 (14.4%)	33 (16.5%)	2.7671	0.2507
Levodopa + COMT inhibitors	95 (13.4%)	19 (14.6%)	48 (12.6%)	28 (14.0%)	0.4386	0.8031
Age > 65 years without cognitive impairment						
Levodopa	345 (48.5%)	42 (32.3%)	187 (49.1%)	116 (58.0%)	20.9235	< 0.0001
Dopamine agonists	180 (25.3%)	22 (16.9%)	99 (26.0%)	59 (29.5%)	6.7851	0.0336
MAO-B inhibitors	101 (14.2%)	15 (11.5%)	62 (16.3%)	24 (12.0%)	2.8932	0.2354
Benzhexol	32 (4.5%)	5 (3.9%)	19 (5.0%)	8 (4.0%)	0.4558	0.7962
Amantadine	69 (9.7%)	8 (6.2%)	44 (11.6%)	17 (8.5%)	3.6800	0.1588
Levodopa + COMT inhibitors	145 (20.4%)	22 (16.9%)	82 (21.5%)	41 (20.5%)	1.2648	0.5313
Wearing off phenomenon						
Add levodopa dose	127 (17.9%)	15 (11.5%)	67 (17.6%)	45 (22.5%)	6.4953	0.0389
Adjust protein diet	181 (25.5%)	18 (13.9%)	87 (22.8%)	76 (38.0%)	27.1973	< 0.0001
Switch from standard levodopa to CR levodopa	260 (36.6%)	30 (23.1%)	140 (36.8%)	90 (45.0%)	16.3361	0.0003
Add COMT inhibitors or MAO-B inhibitors	326 (45.9%)	48 (36.9%)	163 (42.8%)	115 (57.5%)	16.5500	0.0003
Recommend surgical treatment	83 (11.7%)	7 (5.4%)	35 (9.2%)	41 (20.5%)	22.3837	< 0.0001
Peak-dose dyskinesia						
Reduce levodopa dose, add its frequency	296 (41.6%)	7 (28.5%)	156 (40.9%)	103 (51.5%)	17.3687	0.0002
Reduce levodopa dose, add dopamine agonists	319 (44.9%)	33 (25.4%)	168 (44.1%)	118 (59.0%)	36.1891	< 0.0001
Reduce levodopa dose, add COMT inhibitors	249 (35.0%)	33 (25.4%)	129 (33.9%)	87 (43.5%)	11.8497	0.0027
Reduce levodopa dose, add MAO-B inhibitors	204 (28.7%)	23 (17.7%)	101 (26.5%)	80 (40.0%)	21.0749	< 0.0001
Add amantadine	182 (25.6%)	20 (15.4%)	85 (22.3%)	77 (38.5%)	26.7639	< 0.0001
Switch from CR levodopa to standard levodopa	110 (15.5%)	16 (12.3%)	43 (11.3%)	51 (25.5%)	21.4792	< 0.0001

^a^six participants supply insufficient data and not included in the present table

With regard to different class of hospital, we found that all participating hospitals were in agreement on most of the questions, including the treatment of PD patients aged < 65 years without cognitive decline, management of wearing off phenomenon, peak-dose dyskinesia and morning dystonia. They also shared similar opinions on treatment strategies for mental disorder and psychiatric symptoms. Some of them had slight differences in treating constipation and held opposite views on continuous dopaminergic stimulation (CDS). But there were no significant differences in the usage of MAO-B inhibitors and the management of Restless legs syndrome (RLS).

Regarding to financial status of the cities in which the participants were working, we discovered that most of the questions we devised received similar responses—that is, no significant differences in PD treatment and understanding were found among the cities we investigated.

### Guideline’s impact on PD recognition and treatment

Prominent changes have been found. 68.3% of participants agreed with a “low and slow” and dosage titration method as the PD medication principle. In addition to different clinical feature of each patient, 77.7% of participants stressed the necessity of taking ages into account. Specifically, for the treatment of patients aged > 65 years, the top three preferred therapies before the guidelines were levodopa (75.7%), dopamine agonists and MAO-B inhibitors; 29.1% would consider trying a combination of levodopa and catechol-O-methyl transferase (COMT) inhibitors, which have superseded MAO-B inhibitors as the third most common option after the guidelines issued. Patients with wearing off phenomenon were likely to receive more doses of levodopa (52.9%) before the guidelines were issued; now, they would be given COMT inhibitors and MAO-B inhibitors (66.9%) instead. Most participants agreed with a 200-300 mg per day as the recommended starting dose for levodopa treatment. Memantine has been acknowledged as one of the preferred options (57.9%) for treating Parkinson’s disease with dementia (PDD) (χ^2^ (*N* = 717) = 21.1521, *p* < 0.0001) (see Table 2).

**Table 2:** The impact of PD guidelines reference on the selection of medication under specific circumstances

Items	Overall (*n* = 717)	Haven’t read the PD guidelines (*n* = 57)	Have read the PD guidelines (*n* = 660)	χ^2^	*P* value
Age < 65 years without cognitive impairment					
Levodopa	185 (25.8%)	5 (8.8%)	180 (27.3%)	9.3807	0.0022
Dopamine agonists	334 (46.6%)	4 (7.0%)	330 (50.0%)	38.9561	< 0.0001
MAO-B inhibitors	164 (22.9%)	2 (3.5%)	162 (24.6%)	13.1620	0.0003
Benzhexol	49 (6.8%)	0 (0.0%)	49 (7.4%)	4.5422	0.0331
Amantadine	101 (14.1%)	1 (1.8%)	100 (15.2%)	7.7814	0.0053
Levodopa + COMT inhibitors	95 (13.3%)	1 (1.8%)	94 (14.2%)	7.1189	0.0076
Age > 65 years without cognitive impairment					
Levodopa	345 (48.1%)	7 (12.3%)	338 (51.2%)	31.8549	< 0.0001
Dopamine agonists	181 (25.2%)	3 (5.3%)	178 (27.0%)	13.1001	0.0003
MAO-B inhibitors	101 (14.1%)	3 (5.3%)	98 (14.9%)	3.9834	0.0460
Benzhexol	32 (4.5%)	0 (0.0%)	32 (4.9%)	2.8927	0.0890
Amantadine	69 (9.6%)	2 (3.5%)	67 (10.2%)	2.6620	0.1028
Levodopa + COMT inhibitors	145 (20.2%)	1 (1.8%)	144 (21.8%)	13.0918	0.0003
Wearing off phenomenon					
Add levodopa dose	127 (17.7%)	3 (5.3%)	124 (18.8%)	6.5847	0.0103
Adjust protein diet	182 (25.4%)	2 (3.5%)	180 (27.3%)	15.6441	< 0.0001
Switch from standard levodopa to CR levodopa	261 (26.4%)	3 (5.3%)	258 (39.1%)	25.9346	< 0.0001
Add COMT inhibitors or MAO-B inhibitors	327 (45.6%)	5 (8.8%)	322 (28.8%)	33.8682	< 0.0001
Recommend surgical treatment	83 (11.6%)	0 (0.0%)	83 (12.6%)	8.1066	0.0044
Peak-dose dyskinesia					
Reduce levodopa dose, add its frequency	297 (41.4%)	5 (8.8%)	292 (44.2%)	27.2061	< 0.0001
Reduce levodopa dose, add dopamine agonists	319 (44.5%)	7 (12.3%)	312 (47.3%)	26.0137	< 0.0001
Reduce levodopa dose, add COMT inhibitors	251 (35.0%)	5 (8.8%)	246 (37.3%)	18.7324	< 0.0001
Reduce levodopa dose, add MAO-B inhibitors	206 (28.7%)	4 (7.0%)	202 (30.6%)	14.2578	0.0002
Add amantadine	182 (25.4%)	2 (3.5%)	180 (27.3%)	15.6441	< 0.0001
Switch from CR levodopa to standard levodopa	110 (15.3%)	0 (0.0%)	110 (16.7%)	11.2216	0.0008
PD with psychosis (advanced stage)					
Reduce levodopa dose	147 (20.5%)	2 (3.5%)	145 (22.0%)	10.9712	0.0009
Add antipsychotics	321 (44.8%)	11 (19.3%)	310 (47.0%)	16.2481	< 0.0001
PD with visual hallucination and delirium during treatment (ineffective with drug adjustment)					
Clozapine	267 (37.2%)	13 (22.8%)	254 (38.5%)	5.5181	0.0188
Olanzapine	301 (42.0%)	22 (38.6%)	279 (42.3%)	0.2911	0.5895
Quetiapine	301 (42.0%)	9 (15.8%)	292 (44.2%)	17.4394	< 0.0001
Risperidone	85 (11.9%)	3 (5.3%)	82 (12.4%)	2.5749	0.1086
PD with depression					
Tricyclic antidepressants	59 (8.2%)	8 (14.0%)	51 (7.7%)	2.7645	0.0964
SSRIs, e.g. sertraline	487 (67.9%)	39 (68.4%)	448 (67.9%)	0.0071	0.9329
Pramipexole	420 (58.6%)	20 (35.1%)	400 (60.6%)	14.0811	0.0002
PD with dementia (PDD)					
Huperzine A	82 (11.4%)	1 (1.8%)	81 (12.3%)	5.7312	0.0167
Donepezil	346 (48.3%)	13 (22.8%)	333 (50.5%)	16.0620	< 0.0001
Rivastigmine tartrate	275 (38.4%)	7 (12.3%)	268 (40.6%)	17.8047	< 0.0001
Memantine	397 (55.4%)	15 (26.3%)	382 (57.9%)	21.1521	< 0.0001
Traditional Chinese Medicine (TCM), e.g. ginkgo leaf	82 (11.4%)	1 (1.8%)	81 (12.3%)	5.7312	0.0167
PD with periodical limb movement syndrome (PLMS) or restless legs syndrome (RLS)					
Levodopa	153 (21.3%)	6 (10.5%)	147 (22.3%)	4.3130	0.0378
Dopamine agonists	376 (52.4%)	6 (10.5%)	370 (56.1%)	43.6187	< 0.0001
Amantadine	17 (2.4%)	0 (0.0%)	17 (2.6%)	1.5038	0.2201
Benzodiazepines	94 (13.1%)	3 (5.3%)	91 (13.8%)	3.3472	0.0673

We tallied the general satisfaction on the updated guidelines at the end of our investigation. More than 95% of the respondents confirmed its efficiency and application value. Importantly, 36.1% of participants reported a 25–50% of improvement in PD recognition, and 73.5% of them responded ≥25% increased accuracy in diagnosis. These findings reflect the benefit of the wider use of the guidelines.

## Discussion

The purpose of this investigation is to determine at what level our neurologists are on the PD recognition and therapeutic methods. The current situation in China concerning PD management is that this disease is not only treated in neurological department, but non-neurology as well. What’s more, the attending doctors are not simply limited to PD professions, but general neurologist of no PD-treating experience, or even physicians of other departments also. We deem it necessary to gain a thorough and validated situation of status quo on how the PD treatment is really carried out in China and the differences in therapeutic level among doctors of distinguished backgrounds. Supposing that the results of this survey shows significant differences in PD recognition and treatment methods, it is hopefully an optimal guidance for further reformation and improvement in this particular field.

During stage 1, we gathered information of participants involved in this study and classified their backgrounds. Female doctors made up of 57% of the total, and 66% of the samples were in the age group of 35–60 years. With 78% of them selected in first-tier or quasi first-tier cities and 98% worked in tertiary class hospitals, we could determine that this study is highly representative in large public hospitals among capital cities with integrated financial and economic centers in China. During stage 2, we asked the participants to fill in the questionnaire which contains clinical tendency on PD and their personal understanding on certain therapy. We found that the most notable discrepancies came from the feedbacks given by doctors of different specialties. Among which, highly significant differences were seen not only in guideline reference, academic exchanging, researching experience, but were not rare in detailed diagnostic methods like Madopar loading test on suspected patients and MRI application. Situations like these further substantiate our hypothesis that PD treatment in China have not been completely standardized so far. When compared on the ground of distinguished levels of hospitals and cities, we discovered minor differences, indicating the fact that the diagnostic and therapeutic level among hospitals and cities are generally similar, with no significant differences exposed. During stage 3, we discovered an inspirational phenomenon that the treating methods and PD recognition have prominently ameliorated after referring to the CMDS guidelines. Moreover, symptoms presented on PD patients have been significantly improved as well, as over half of the doctors surveyed reported improvements in their patients. Specifically, 36.8% of the participants confirmed an over 50% of improvements during clinical practice; while 62.1 and 63.1% of participants affirmed a reduction in patients with wearing off phenomenon and peak-dose dyskinesia, respectively. In short, the guideline is markedly beneficial to reasonable PD medications strategy, and hopefully, its dissemination will greatly enhance the standard of our PD management.

Compared to the guidelines issued before represented by MDS, etc., CMDS guidelines has a relatively short history. When first issued in 2006, it has been updated regularly and remained one of the young members among the family of PD guidelines worldwide. Its localization strategy in optimizing and standardizing management of PD, especially its emphasis about “low and slow”, have covered a large range of patients in need, and is conducive to disease management in a prolonged period [5, 6]. Inspirationally, we found that the clinical guideline is already helping the standardization of practice via improving the level of clinicians. It not only improved the diagnostic accuracy, but decreased the motor complications as well, which is in consistent with another study we have done recently [6].

However, since the survey was performed on doctors from tertiary class hospitals only, with no data gathered from the patients, we have not drawn any objective statistics that could directly show the improvement of the effectiveness of PD management and the health condition on patients’ perspective. Further researches need to be conducted in order to make up for this deficiency.

## Conclusions

In general, the CMDS guidelines has demonstrated a clear impact on therapeutic strategies for PD, and the clinical management of PD in China could be improved considerably if the deficiencies revealed in this study could be effectively addressed. Hopefully, the wider dissemination of the CMDS guidelines will greatly enhance the standard of China’s PD management continuatively in the long run.

## Abbreviations

AAN: American Academy of Neurology
CDS: Continuous dopaminergic stimulation
CMDS: Chinese Parkinson’s Disease & Movement Disorders Society
EFNS: European Federation of Neurological Societies
GPs: General practitioners
MAO-B: Monoamine oxidase BCOMT: catechol-O-methyl transferase
MDS: Movement Disorder Society
NICE: UK’s National Institute for Health and Care Excellence
PD: Parkinson’s disease
PDD: Parkinson’s disease with dementia
RLS: Restless legs syndrome
